# Supplementary material for: Factors associated with persistently high-cost health care utilization for musculoskeletal pain
Source: PLoS One. 2019 Nov 11;14(11):e0225125. doi: 10.1371/journal.pone.0225125 (PMC6844454; doi:10.1371/journal.pone.0225125)
Supplement: S1 Appendix — (DOCX) [file pone.0225125.s001.docx]

S1 APPENDIX

Definition and Analysis Coding of Categorical Variables

Sex

1. Male (reference)
2. Female

Race

1. White
2. Black
3. American Indian/Alaska Native
4. Asian
5. Native Hawaiian/Pacific Islander
6. Multiple race reported

Race groups were collapsed for analysis:

1. White (reference)
2. Black
3. Other (American Indian/Alaska Native, Asian, Native Hawaiian/Pacific Islander, multiple races).

Ethnicity

1. Hispanic
2. Non-Hispanic (reference)

Years of Education

1. No degree
2. GED
3. High school diploma
4. Bachelor’s degree
5. Master’s degree
6. Doctorate degree
7. Other degree

Years of education was collapsed for analysis:

1. High school diploma or less (reference)
2. Bachelor’s degree or more.

Smoking Status

1. Yes
2. No (reference)

Poverty Category

1. Negative or Poor: Persons in families with income less than or equal to the poverty line and includes those who reported negative income.
2. Near-poor: Persons in families with income over the poverty line through 125 percent of the poverty line.
3. Low income: Persons in families with income over 125 percent through 200 percent of the poverty line.
4. Middle income: Persons in families with income over 200 percent through 400 percent of the poverty line.
5. High income: Persons in families with income over 400 percent of the poverty line.

Poverty category was collapsed for analysis:

1. Poor/Negative/Near Poor
2. Low Income
3. Middle Income
4. High Income (reference)

Employment Status (Round 1)

1. Employed
2. Job to return to
3. Job during the reference period
4. Not employed

Employment status was collapsed for analysis:

1. Employed (Employed, Job to return to, Job during reference period) (reference)
2. Unemployed (not employed)

Metropolitan Statistical Area (MSA)

1. Non-MSA
2. MSA (reference)

Census region

1. Northeast (reference)
2. Midwest
3. South
4. West

Days of Work Missed due to Illness

The number of times the respondent lost a half-day or more from work because of illness, injury, or mental or emotional problems were recorded for each of the 5 rounds in Year 1 and summed for total days of work missed.

Days of work missed was dichotomized for analysis:

1. no missed days (reference)
2. one of more days missed

Pain Interference

Pain interference with work and daily activities was assessed using the following SF-12 question from the Adult SAQ: “During past 4 weeks, how much has pain interfered with normal work outside the home and housework?

Possible responses include:

1. None
2. A little bit
3. Moderately
4. Quite a bit
5. Extremely

Pain interference was dichotomized for analysis:

1. None/A little bit/Moderately (reference)
2. Quite a bit/Extremely

Perceived Health Status

Perceived health status was reported as fair/poor (reference) or excellent/very good/good.

1. Excellent
2. Very good
3. Good
4. Fair
5. Poor

Perceived health status was dichotomized for analysis:

1. Excellent/Very good/Good (reference)
2. Fair/Poor

Perceived Mental Health Status

Perceived mental health status was reported as fair/poor (reference) or excellent/very good/good.

1. Excellent
2. Very good
3. Good
4. Fair
5. Poor

Perceived health status was dichotomized for analysis:

1. Excellent/Very good/Good (reference)
2. Fair/Poor

Can Overcome Ills

The Adult SAQ includes a question that ascertains whether the respondent believes they can overcome illness without help from a medically trained person.

1. Disagree strongly
2. Disagree somewhat
3. Uncertain
4. Agree somewhat
5. Agree strongly

Overcome ills was dichotomized for analysis:

1. Disagree strongly/Disagree somewhat
2. Uncertain/Agree somewhat/Agree strongly (reference)

Usual Healthcare Provider

1. Yes
2. No (reference)

Health Insurance

MEPS provides information on monthly payer status for each of the following:

1. TRICARE - Health care program of the United States Department of Defense Military Health System. Tricare provides civilian health benefits for U.S Armed Forces military personnel, military retirees, and their dependents.
2. Medicare - Federal government-sponsored health insurance for adults aged 65 and older, younger people with some disability status, and people with end stage renal disease and amyotrophic lateral sclerosis.
3. Medicaid/State Children’s Health Insurance Program (SCHIP) - federal and state-sponsored health insurance program for eligible low-income adults, children, pregnant women, elderly adults and people with disabilities. Medicaid is administered by states, according to federal requirements. The program is funded jointly by states and the federal government.
4. Private insurance – Health insurance policy purchased by an individual or employer from a private health insurance company.

MEPS also includes summary measures that indicate whether or not a person has any insurance in a month. Each respondent was categorized as being privately insured all year, publicly insured all year, uninsured part of the year and either privately or publicly insured the remainder or uninsured all year. Tricare, Medicare, and Medicaid/SCHIP are forms of public insurance.

Insurance status was collapsed for analysis:

1. Privately insured all year
2. Publicly insured all year
3. Uninsured part of the year and either privately or publicly insured the remainder, or uninsured all year (reference)

Diagnosis

1. Diseases of The Musculoskeletal System and Connective Tissue diagnosis/diagnoses only (reference)
2. Musculoskeletal Injury diagnosis with or without Diseases of the Musculoskeletal System and Connective Tissue diagnosis/diagnoses

Comorbidity (Charlson Comorbidity Index)

1. Myocardial infarction
2. Congestive heart failure
3. Peripheral vascular disorders
4. Cerebrovascular disease
5. Dementia
6. Chronic pulmonary disease
7. Rheumatic disease
8. Peptic ulcer disease
9. Mild liver disease
10. Diabetes without chronic complication
11. Diabetes with chronic complication
12. Hemiplegia or paraplegia
13. Renal disease
14. Any malignancy, including lymphoma and leukemia, except malignant neoplasm of skin
15. Moderate or severe liver disease
16. Metastatic solid tumor
17. AIDS/HIV
