# Supplementary material for: Factors associated with persistently high-cost health care utilization for musculoskeletal pain
Source: PLoS One. 2019 Nov 11;14(11):e0225125. doi: 10.1371/journal.pone.0225125 (PMC6844454; doi:10.1371/journal.pone.0225125)
Supplement: S1 Table — (DOCX) [file pone.0225125.s002.docx]

**S1 Table.** List of musculoskeletal ICD-9 code diagnoses

| Diagnoses included in the analysis |
| --- |
| 715 : Osteoarthrosis and allied disorders  716 : Other and unspecified arthroplasties  717 : Internal derangement of knee  718 : Other derangement of joint  719 : Other and unspecified disorders of joint  720 : Ankylosing spondylitis and other inflammatory spondylopathies  721 : Spondylosis and allied disorders  722 : Intervertebral disc disorders  723 : Other disorder of cervical region  724 : Other and unspecified disorders of back  725 : Polymyalgia rheumatica  726 : Peripheral enthesopathies and allied syndromes  727 : Synovitis and tenosynovitis  728 : Disorders of muscle, ligament, and fascia  729 : Other disorders of soft tissue  730 : Acute osteomyelitis  731 : Osteitis deformans and osteopathies associated with other disorders classified elsewhere  732 : Osteochondropathies  733 : Other disorders of bone and cartilage (Osteoporosis; pathologic fracture, cyst, necrosis of bone, malunion and nonunion of fracture)  734 : Flat foot  735 : Acquired deformities of toe  736 : Acquired deformities of forearm  737 : Curvature of spine  738 : Other acquired deformity (of musculoskeletal system), spondylolisthesis  739 : Nonallopathic lesions, not elsewhere classified  805 : Fracture of vertebral column without mention of spinal cord injury  808 : Fracture of pelvis (Acetabulum, closed)  809 : Ill-defined fractures of bones and trunk  810 : Fracture of clavicle (closed)  811 : Fracture of scapula (closed)  812 : Fracture of humerus (Upper end, closed)  813 : Fracture of radius and ulna (Upper end, closed)  814 : Fracture of carpal bone(s) (Closed)  815 : Fracture of metacarpal bone(s) (Closed)  816 : Fracture of one or more phalanges of hand (Closed)  817 : Multiple fractures of hand bones  818 : Ill-defined fractures of upper limb  820 : Fracture of neck of femur (transcervical fracture, closed)  821 : Fracture of other and unspecified parts of femur (Shaft or unspecified part, closed)  822 : Fracture of patella  823 : Fracture of tibia and fibula, upper end (closed)  824 : Fracture of ankle  825 : Fracture of one or more tarsal and metatarsal bones  826 : Fracture of one or more phalanges of foot  827 : Other, multiple, and ill-defined fractures of lower limb  829 : Fractures of unspecified bones  831 : Dislocation of shoulder  832 : Dislocation of elbow  833 : Dislocation of wrist  834 : Dislocation of finger  835 : Dislocation of hip  836 : Dislocation of knee  837 : Dislocation of ankle  838 : Dislocation of foot  839 : Other, multiple, and ill-defined dislocations  840 : Sprains and strains of shoulder and upper arm  841 : Sprains and strains of elbow and forearm  842 : Sprains and strains of wrist and hand  843 : Sprains and strains of hip and thigh  844 : Sprains and strains of knee and leg  845 : Sprains and strains of ankle and foot  846 : Sprains and strains of sacroiliac region  847 : Sprains and strains of other and unspecified parts of back  848 : Other and ill-defined sprains and strains  922 : Contusion of trunk  923 : Contusion of upper limb  924 : Contusion of lower limb and of other and unspecified sites  954 : Injury to other nerve(s) of trunk, excluding shoulder and pelvic girdles  955 : Injury to peripheral nerve(s) of shoulder girdle and upper limb  956 : Injury to peripheral nerve(s) of pelvic girdle and lower limb  959 : Injury, other and unspecified (to musculoskeletal system) |
| Diagnoses excluded from the analysis |
| 135 : Sarcoidosis  170 : Malignant neoplasm of bone and articular cartilage  171 : Malignant neoplasm of connective and other soft tissue  198 : Secondary malignant neoplasm of bone and bone marrow  203 : Multiple myeloma and immunoproliferative neoplasms  213 : Benign neoplasm of bone and articular cartilage  215 : Other benign neoplasm of connective and other soft tissue  238 : Neoplasm of uncertain behavior of other and unspecified sites and tissues; Connective and other soft tissue; Bone soft tissue and skin  239.2 : Neoplasms of unspecified nature; Bone soft tissue and skin  274 : Gout; Gouty arthroplathy  354 : Mononeuritis of upper limb and mononeuritis multiplex  710 : Diffuse diseases of connective tissue  711 : Arthropathy associated with infections  712 : Crystal arthropathies  713 : Arthropathy associated with other disorders classified elsewhere  714 : Rheumatoid arthritis and other inflammatory polyarthropathies  741 : Spina bifida  754 : Certain congenital musculoskeletal deformities  755 : Other congenital anomalies of limbs (Polydactyly)  756 : Other congenital musculoskeletal anomalies  806 : Fracture of vertebral column with mention of spinal cord injury  807 : Fracture of vertebral column with mention of spinal cord injury  819 : Multiple fractures involving both upper limbs, and upper limb with rib(s) and sternum  875 : Open wound of chest (wall)  876 : Open wound of back  877 : Open wound of buttock  879 : Open wound of other and unspecified sites (except limbs)  880 : Open wound of shoulder and upper arm  881 : Open wound of elbow, forearm, and wrist  882 : Open wound of hand except finger(s) alone  883 : Open wound of finger(s)  884 : Multiple and unspecified open wound of upper limb  885 : Traumatic amputation of thumb  886 : Traumatic amputation of other finger(s)  887 : Traumatic amputation of arm and hand (complete) (partial)  890 : Open wound of hip and thigh  891 : Open wound of knee, leg [except thigh], and ankle  892 : Open wound of foot except toe(s) alone  893 : Open wound of toe(s)  894 : Multiple and unspecified open wound of lower limb  895 : Traumatic amputation of toe(s)  896 : Traumatic amputation of foot (complete) (partial)  897 : Traumatic amputation of leg(s) (complete) (partial)  926 : Crushing injury of trunk  927 : Crushing injury of upper limb  928 : Crushing injury of lower limb  929 : Crushing injury of multiple and unspecified sites  996 : Complications peculiar to certain specified procedures  V43.6 : Organ or tissue replaced by other means (joint)  V54 : Other orthopaedic aftercare  V67 : Follow-up examination, following surgery |
