# Supplementary material for: Factors associated with persistently high-cost health care utilization for musculoskeletal pain
Source: PLoS One. 2019 Nov 11;14(11):e0225125. doi: 10.1371/journal.pone.0225125 (PMC6844454; doi:10.1371/journal.pone.0225125)
Supplement: S2 Table — a Data are mean ± standard error of mean (range). b Unweighted sample size. PCS = physical component subscale of the SF-12, MCS = mental component subscale of the SF-12. (DOCX) [file pone.0225125.s003.docx]

**S2 Table.** Weighted means for demographic and health-related information that differed significantly between groups.

| Variable^a^ | Use of proxy to complete SAQ (n=1,223) | Self-completion of SAQ  (n=13,332) | p-value |
| --- | --- | --- | --- |
| Age, yrs | 58.9 ± 0.3  (18-85) | 54.0 ± 0.6  (18-85) | <.001 |
| Body mass index | 27.5 ± 0.2  (14.1-77.3) | 28.9 ± 0.1  (9.4-82.1) | <.001 |
| Charlson comorbidity index | 0.8 ± 0.01  (0-9) | 0.6 ± 0.01  (0-11) | <.001 |
| PCS | 38.4 ± 0.5  (7.3-64.3) | 43.7 ± 0.2  (5.9-69.2) | <.001 |
| MCS | 47.4 ± 0.2  (11.2-74.8) | 49.7 ± 0.1  (5.2 – 78.0) | <.001 |
| General psychological distress | 5.7 ± 0.2  (0-24) | 4.4 ± 0.1  (0-24) | <.001 |
| Depression | 1.3 ± 0.1  (0-6) | 1.0 ± 0.2  (0-6) | <.001 |
| Total musculoskeletal conditions | 1.6 ± 0.04  (1-8) | 1.8 ± 0.02  (1-10) | <.001 |

^a^ Data are mean ± standard error of mean (range)

^b^ Unweighted sample size.

PCS = physical component subscale of the SF-12, MCS = mental component subscale of the SF-12.
