# Supplementary material for: Factors associated with persistently high-cost health care utilization for musculoskeletal pain
Source: PLoS One. 2019 Nov 11;14(11):e0225125. doi: 10.1371/journal.pone.0225125 (PMC6844454; doi:10.1371/journal.pone.0225125)
Supplement: S3 Table — a Data are sample size (weighted population percentage estimates). b Unweighted sample size. (DOCX) [file pone.0225125.s004.docx]

**S3 Table.** Sample distributions with weighted population percentage estimates for demographic and health-related information that differed significantly between groups

| Variable^a^ | | Use of proxy to complete SAQ (n=1,223) ^b^ | Self-completion of SAQ  (n=13,332) ^b^ | p-value |
| --- | --- | --- | --- | --- |
| Sex | Male | 665 (58.0) | 4,888 (39.5) | <.001 |
|  | Female | 558 (41.8) | 8444 (60.5) |  |
| Race | White | 791 (78.9) | 9,832 (85.4) | <.001 |
|  | Black | 253 (11.3) | 2,546 (9.7) |  |
|  | Other | 174 (9.8) | 944 (4.9) |  |
| Ethnicity | Hispanic | 272 (11.7) | 2,254 (8.5) | <.001 |
|  | Non-Hispanic | 951 (88.3) | 11,078 (91.5) |  |
| Poverty category | Poor or Near Poor | 356  (19.0) | 3,179 (16.0) | <.001 |
|  | Low Income | 241 (20.0) | 2,038 (13.4) |  |
|  | Middle Income | 344 (29.7) | 3,841 (28.6) |  |
|  | High Income | 282 (31.4) | 4,274 (42.0) |  |
| Education | High school diploma or less | 999 (74.6) | 8,473 (58.0) | <.001 |
|  | Some college/college degree | 224 (25.4) | 4,859 (42.0) |  |
| Employment | Employed or have a job to return to | 437 (39.7) | 6,921 (55.2) | <.001 |
|  | Unemployed | 779 (60.3) | 6,365 (44.8) |  |
| Pain interference | Not at all, a little bit, or moderately | 737 (63.4) | 9,573 (76.0) | <.001 |
|  | Quite a bit or extremely | 465 (36.6) | 3,580 (24.0) |  |
| Ability to overcome | Disagree strongly, disagree somewhat or uncertain | 1,064 (86.9) | 10,856 (82.4) | 0.003 |
|  | Agree somewhat or agree strongly | 139 (13.1) | 2,097 (17.6) |  |
| Insurance | Uninsured | 220 (17.1) | 2,396 (14.8) | <.001 |
|  | Public Insurance | 540 (37.2) | 4,059 (26.1) |  |
|  | Private Insurance | 463 (45.7) | 6,877 (59.1) |  |
| Physical health | Excellent, very good, or good | 719 (62.4) | 9,584 (76.7) | <.001 |
|  | Fair or poor | 500 (37.6) | 3,736 (23.3) |  |
| Mental health | Excellent, very good, or good | 956 (80.5) | 11,752 (90.2) | <.001 |
|  | Fair or poor | 263 (19.4) | 1,569 (9.8) |  |
| Metropolitan statistical area (MSA) | Non-MSA | 229 (20.9) | 2,202 (17.6) | 0.03 |
|  | MSA | 994 (79.13) | 11,129 (82.4) |  |

^a^ Data are sample size (weighted population percentage estimates)

^b^ Unweighted sample size
