# Supplementary material for: Factors associated with persistently high-cost health care utilization for musculoskeletal pain
Source: PLoS One. 2019 Nov 11;14(11):e0225125. doi: 10.1371/journal.pone.0225125 (PMC6844454; doi:10.1371/journal.pone.0225125)
Supplement: S4 Table — (DOCX) [file pone.0225125.s005.docx]

**S4 Table.** Unweighted frequency table of diseases of the musculoskeletal system and connective tissue ICD-9 codes.

| ICD-9 diagnostic category | ICD-9 code | Low  (n=1504) | Medium  (n=10,983) | High  (n=498) | Total |
| --- | --- | --- | --- | --- | --- |
| Diseases of the musculoskeletal system and connective tissue | 719 | 433 | 3,470 | 206 | 4,109 |
|  | 724 | 260 | 2,403 | 207 | 2,870 |
|  | 716 | 240 | 2,176 | 179 | 2,595 |
|  | 715 | 140 | 1,907 | 169 | 2,216 |
|  | 729 | 165 | 1,440 | 154 | 1,759 |
|  | 733 | 65 | 804 | 31 | 900 |
|  | 722 | 28 | 726 | 117 | 871 |
|  | 728 | 58 | 562 | 57 | 677 |
|  | 726 | 44 | 527 | 35 | 606 |
|  | 723 | 37 | 425 | 40 | 502 |
|  | 727 | 16 | 331 | 22 | 369 |
|  | 734 | 6 | 183 | 29 | 218 |
|  | 737 | 4 | 80 | 8 | 92 |
|  | 721 | 6 | 68 | 6 | 80 |
|  | 717 | 2 | 49 | 8 | 59 |
|  | 735 | 1 | 47 | 2 | 50 |
|  | 738 | 2 | 40 | 7 | 49 |
|  | 718 | 1 | 32 | 3 | 36 |
|  | 736 | 0 | 33 | 3 | 36 |
|  | 730 | 2 | 15 | 2 | 19 |
|  | 725 | 1 | 6 | 3 | 10 |
