# Supplementary material for: Factors associated with persistently high-cost health care utilization for musculoskeletal pain
Source: PLoS One. 2019 Nov 11;14(11):e0225125. doi: 10.1371/journal.pone.0225125 (PMC6844454; doi:10.1371/journal.pone.0225125)
Supplement: S5 Table — (DOCX) [file pone.0225125.s006.docx]

**S5 Table.** Unweighted frequency table of musculoskeletal injury ICD-9 codes.

| ICD-9 diagnostic  category | ICD-9  code | Low  (n=1504) | Medium (n=10,983) | High  (n=498) | Total |
| --- | --- | --- | --- | --- | --- |
| Musculoskeletal injuries | 959 | 86 | 1,076 | 96 | 1,258 |
|  | 847 | 40 | 354 | 16 | 410 |
|  | 845 | 35 | 318 | 8 | 361 |
|  | 840 | 10 | 286 | 27 | 323 |
|  | 848 | 28 | 210 | 11 | 249 |
|  | 924 | 17 | 206 | 8 | 231 |
|  | 844 | 20 | 190 | 9 | 219 |
|  | 836 | 2 | 133 | 9 | 144 |
|  | 842 | 17 | 109 | 2 | 128 |
|  | 825 | 8 | 107 | 6 | 121 |
|  | 839 | 8 | 87 | 4 | 99 |
|  | 826 | 9 | 79 | 3 | 91 |
|  | 805 | 3 | 71 | 14 | 88 |
|  | 814 | 2 | 74 | 6 | 82 |
|  | 824 | 2 | 75 | 4 | 81 |
|  | 923 | 6 | 69 | 5 | 80 |
|  | 922 | 6 | 63 | 3 | 72 |
|  | 816 | 5 | 62 | 0 | 67 |
|  | 843 | 3 | 49 | 4 | 56 |
|  | 815 | 2 | 47 | 2 | 51 |
|  | 822 | 0 | 44 | 5 | 49 |
|  | 831 | 2 | 46 | 0 | 48 |
|  | 812 | 0 | 46 | 0 | 46 |
|  | 818 | 0 | 43 | 3 | 46 |
|  | 827 | 4 | 34 | 8 | 46 |
|  | 820 | 0 | 27 | 3 | 30 |
|  | 810 | 1 | 21 | 0 | 22 |
|  | 835 | 0 | 15 | 7 | 22 |
|  | 846 | 3 | 18 | 1 | 22 |
|  | 829 | 1 | 18 | 2 | 21 |
|  | 837 | 1 | 9 | 5 | 15 |
|  | 834 | 2 | 11 | 1 | 14 |
|  | 808 | 0 | 10 | 1 | 11 |
|  | 813 | 0 | 9 | 0 | 9 |
|  | 821 | 0 | 5 | 3 | 8 |
|  | 823 | 0 | 7 | 1 | 8 |
|  | 832 | 0 | 6 | 0 | 6 |
|  | 841 | 2 | 3 | 0 | 5 |
|  | 811 | 0 | 4 | 0 | 4 |
|  | 838 | 0 | 4 | 0 | 4 |
|  | 837 | 0 | 1 | 0 | 1 |
