# Supplementary material for: Factors associated with persistently high-cost health care utilization for musculoskeletal pain
Source: PLoS One. 2019 Nov 11;14(11):e0225125. doi: 10.1371/journal.pone.0225125 (PMC6844454; doi:10.1371/journal.pone.0225125)
Supplement: S6 Table — a Model fit statistics: Generalized Logit model Wald Chi-square test = 20.20, p < .001. b Adjusted odds ratios reflect odds of being classified as HIGH compared to the reference classification. For continuous variables, values represent odds of being classified as HIGH compared to the reference classification for every one unit increase in the variable. Low = annual pain-related expenditures in the lowest 15%; Medium = annual pain-related expenditures between 15–85%; PCS = physical component subscale of the SF-12, MCS = mental component subscale of the SF-12. (DOCX) [file pone.0225125.s007.docx]

**S6 Table.** Results of fully-adjusted multivariable logistic regression model sensitivity analyses (10% and 20% expenditure percentile criterion)*^a^

| **Variable** | **Reference classification** | **10% Threshold** | | | **20% Threshold** | | |
| --- | --- | --- | --- | --- | --- | --- | --- |
|  |  | **Adjusted odds ratio^b^** | **95% CI** | | **Adjusted odds ratio^b^** | **95% CI** |  |
|  |  |  | **Lower** | **Upper** |  | **Lower** | **Upper** |
| Age | Low | 1.042 | 1.057 | 1.028 | 1.011 | 1.020 | 1.002 |
|  | Medium | 1.031 | 1.044 | 1.017 | 1.003 | 1.012 | 0.994 |
| Sex (Female vs male) | Low | 0.665 | 0.991 | 0.447 | 1.027 | 1.312 | 0.803 |
|  | Medium | 0.746 | 1.082 | 0.515 | 1.003 | 1.266 | 0.794 |
| Race (Black vs white) | Low | 0.877 | 1.337 | 0.575 | 0.587 | 0.822 | 0.419 |
|  | Medium | 1.122 | 1.684 | 0.749 | 0.789 | 1.083 | 0.575 |
| Race (Other vs white) | Low | 0.647 | 1.779 | 0.235 | 0.751 | 1.274 | 0.442 |
|  | Medium | 0.669 | 1.792 | 0.249 | 0.813 | 1.297 | 0.510 |
| Ethnicity (Hispanic vs non-Hispanic) | Low | 1.362 | 2.387 | 0.778 | 0.922 | 1.377 | 0.617 |
|  | Medium | 1.555 | 2.688 | 0.899 | 1.028 | 1.504 | 0.703 |
| Education (Greater than high school vs high school or less) | Low | 1.439 | 2.273 | 0.912 | 1.357 | 1.818 | 1.012 |
|  | Medium | 1.170 | 1.742 | 0.785 | 1.230 | 1.582 | 0.956 |
| Poverty level (Low income vs poor or near poor) | Low | 0.664 | 1.238 | 0.356 | 0.528 | 0.800 | 0.349 |
|  | Medium | 0.759 | 1.383 | 0.417 | 0.651 | 0.924 | 0.458 |
| Poverty level (Middle income vs poor or near poor) | Low | 1.172 | 2.146 | 0.640 | 0.763 | 1.174 | 0.496 |
|  | Medium | 1.089 | 1.908 | 0.621 | 0.792 | 1.124 | 0.558 |
| Poverty level (High income vs poor or near poor) | Low | 1.276 | 2.681 | 0.606 | 1.255 | 1.980 | 0.794 |
|  | Medium | 0.872 | 1.678 | 0.453 | 1.085 | 1.555 | 0.756 |
| Employment (Unemployed vs employed) | Low | 1.045 | 1.742 | 0.626 | 1.211 | 1.730 | 0.847 |
|  | Medium | 0.968 | 1.558 | 0.602 | 1.186 | 1.650 | 0.853 |
| Metropolitan Statistical Area (MSA) (Non-MSA vs MSA) | Low | 0.631 | 1.033 | 0.385 | 0.670 | 1.009 | 0.445 |
|  | Medium | 0.647 | 1.017 | 0.412 | 0.733 | 1.020 | 0.526 |
| Census region (Midwest vs Northeast) | Low | 0.582 | 1.127 | 0.300 | 0.729 | 1.105 | 0.481 |
|  | Medium | 0.792 | 1.479 | 0.425 | 0.873 | 1.238 | 0.615 |
| Census region (South vs Northeast) | Low | 0.454 | 0.882 | 0.233 | 0.651 | 0.988 | 0.429 |
|  | Medium | 0.582 | 1.092 | 0.311 | 0.784 | 1.124 | 0.548 |
| Census region (West vs Northeast) | Low | 0.574 | 1.089 | 0.303 | 0.855 | 1.314 | 0.557 |
|  | Medium | 0.730 | 1.355 | 0.394 | 1.075 | 1.553 | 0.744 |
| Charlson Comorbidity Index (CCI) | Low | 1.005 | 1.190 | 0.847 | 0.961 | 1.091 | 0.845 |
|  | Medium | 1.013 | 1.160 | 0.883 | 0.945 | 1.047 | 0.854 |
| Insurance (Public vs uninsured) | Low | 1.742 | 3.367 | 0.902 | 2.004 | 3.086 | 1.302 |
|  | Medium | 1.712 | 3.155 | 0.929 | 1.689 | 2.481 | 1.147 |
| Insurance (Private vs uninsured) | Low | 2.506 | 5.263 | 1.195 | 2.463 | 3.704 | 1.637 |
|  | Medium | 1.965 | 3.922 | 0.985 | 1.730 | 2.519 | 1.188 |
| Diagnosis type (Injury vs disease only) | Low | 1.745 | 2.625 | 1.163 | 1.502 | 1.927 | 1.168 |
|  | Medium | 1.012 | 1.439 | 0.712 | 1.045 | 1.305 | 0.837 |
| Total musculoskeletal conditions | Low | 3.257 | 4.049 | 2.618 | 3.021 | 3.497 | 2.597 |
|  | Medium | 1.326 | 1.486 | 1.183 | 1.376 | 1.508 | 1.253 |
| Missed work days in Year 1 (missed ≥1-day vs no missed days) | Low | 2.577 | 4.975 | 1.333 | 2.618 | 3.937 | 1.739 |
|  | Medium | 1.499 | 2.653 | 0.847 | 1.560 | 2.193 | 1.111 |
| PCS | Low | 0.959 | 0.981 | 0.936 | 0.968 | 0.983 | 0.952 |
|  | Medium | 0.963 | 0.983 | 0.944 | 0.975 | 0.987 | 0.962 |
| MCS | Low | 0.979 | 1.006 | 0.954 | 0.990 | 1.008 | 0.973 |
|  | Medium | 0.980 | 1.004 | 0.957 | 0.987 | 1.002 | 0.974 |
| Pain interference (Moderate, quite a bit or extreme vs a little bit or none) | Low | 2.088 | 3.610 | 1.205 | 1.590 | 2.237 | 1.133 |
|  | Medium | 1.247 | 2.079 | 0.746 | 1.189 | 1.605 | 0.881 |
| General psychological distress | Low | 0.976 | 1.041 | 0.915 | 0.979 | 1.024 | 0.937 |
|  | Medium | 1.004 | 1.064 | 0.948 | 0.990 | 1.027 | 0.955 |
| Depression | Low | 1.016 | 1.235 | 0.838 | 1.066 | 1.215 | 0.935 |
|  | Medium | 0.968 | 1.148 | 0.816 | 1.048 | 1.167 | 0.943 |
| Perceived health status (Fair or poor vs good, very good or excellent) | Low | 0.824 | 1.304 | 0.522 | 0.873 | 1.195 | 0.637 |
|  | Medium | 1.116 | 1.675 | 0.743 | 1.185 | 1.553 | 0.903 |
| Perceived mental health status (Fair or poor vs good, very good or excellent) | Low | 0.998 | 1.721 | 0.578 | 1.377 | 2.165 | 0.876 |
|  | Medium | 0.751 | 1.161 | 0.485 | 0.921 | 1.325 | 0.640 |
| Total prescription medications in Year 1 | Low | 1.285 | 1.387 | 1.190 | 1.477 | 1.575 | 1.387 |
|  | Medium | 1.175 | 1.238 | 1.115 | 1.208 | 1.252 | 1.166 |

^a^ Model fit statistics: Generalized Logit model Wald Chi-square test = 20.20, p<.001.

^b^ Adjusted odds ratios reflect odds of being classified as HIGH compared to the reference classification. For continuous variables, values represent odds of being classified as HIGH compared to the reference classification for every one unit increase in the variable.

Low = annual pain-related expenditures in the lowest 15%; Medium = annual pain-related expenditures between 15-85%; PCS = physical component subscale of the SF-12, MCS = mental component subscale of the SF-12.
