# Supplementary material for: Factors associated with persistently high-cost health care utilization for musculoskeletal pain
Source: PLoS One. 2019 Nov 11;14(11):e0225125. doi: 10.1371/journal.pone.0225125 (PMC6844454; doi:10.1371/journal.pone.0225125)
Supplement: Attachment — Submitted filename: PLoS Response letter_Final.docx [file pone.0225125.s008.docx]

PONE-D-19-18187
Factors Associated with Persistently High-Cost Health Care Utilization for Musculoskeletal Pain
PLOS ONE

Additional Editor Comments:

Overall this is an interesting and valuable study. I noted several aspects of the methods and analyses that require attention to ensure clear interpretation from readers, and potential future replication of the study.

1. Please justify excluding cases with a proxy response for the adult SAQ (e.g., is it not validated for a proxy response?), and why you chose not to use imputation methods to include cases with incomplete SAQ responses.

**Thank you for the opportunity to clarify this important methodological decision. The SAQ included questions related to physical and mental functioning, depression and general psychological distress. As these are inherently subjective components of individual functioning, we felt that proxy estimates would not be appropriate for the purposes of this study and could impact the validity of the results. Indeed, prior studies (e.g., Elliott, et al., Health Serv Res 2008; Roydhouse, et al., Health Serv Res, 2018; Roydhouse and Wilson, Qual Life Res, 2017) have called into question the use of proxy responses to appropriately assess subjective measures like quality of life. For this reason, we excluded individuals that had a proxy response for the adult SAQ. We have included additional justification for our methods in the revision (lines 82-88, 511-519)**

**Approximately 3% of the eligible sample had incomplete SAQ responses. We used SAQ survey weights (variable: SAQWEIGHT; provided in the MEPS data files) that adjust for SAQ non-response. For each year, the sum of these weights is equal to that year’s civilian, non-institutionalized U.S. population aged 18 and older. Importantly, we did not “drop” any observation from the analytic set, but rather used flags to denote the final analytic sample and appropriate survey procedures to produce unbiased regression estimates. We don’t believe imputation methods or additional statistical adjustment for non-response are necessary, since these weights already account for information that is not available for individuals that did not complete the SAQ.**

2. The STROBE chart should be included in the body of the paper. On this figure it is not clear what data are being shown in the parentheses.

**A revised STROBE chart is now included in the body of the paper. The data in parentheses represented the weighted population based on the sample available at each step. However, we agree this can be confusing for readers, so we now only include the raw sample number at each step.**

3. Can you please explain the “condition-event crosswalk” in more detail.

**The condition event crosswalk is a numeric variable that allows conditions in the MEPS condition files to be matched with their associated medical events (i.e., treatments) in the MEPS event files. This is the method for linking conditions and events provided by AHRQ for MEPS analyses. Additional detail is now provided in the methods.**

4. How is “poverty category” calculated or defined?

**This information is now listed for readers in a new appendix.**

5. To improve clarity for an international audience can a little more detail be provided on the different types of health insurance?

**Thank you for this suggestion. In the revised manuscript we have included supplementary material that provides more detailed information on the categorical variables in the analysis. The supplementary material should address multiple Editor and Reviewer comments, and includes more information on types of health insurance. We intend that this supplementary material will be available to readers.**

6. The analyses used multinomial logistic regression, but did you check whether the outcome was suitable for ordinal regression? Assuming that it was not ordinal, can you also indicate whether the assumption of the independence of irrelevant alternatives was met. Also, the results report odds ratios, but a multinomial logistic regression yields a risk ratio. Please check and clarify.

**We initially examined the appropriateness of both multinomial and ordinal regression. The data did not meet the parallel lines assumption, nor did it meet the assumption of the independence of irrelevant alternatives. Given that, we chose to report results of the multinomial logit since it produces a separate odds ratio for each category rather than an ordinal logit which makes the assumption that the effect of going from category 1 to category 2 is the same as going from category 2 to category 3. Multinomial logit isn’t as restrictive as ordinal logit, and it is more interpretable in this context**. **Thank you for pointing out our error in reporting the risk ratio parameter. We have changed the relevant labels throughout the manuscript.**

7. The results should include an analysis of differences between those excluded from analysis due to loss to follow up. This is particularly relevant given that a loose statement on potential biases due to participant exclusion in the discussion (lines 444-446). If these characteristics were analysed you could comment on what those biases are.

**In this analysis, those who were not in-scope for the entire panel, or did not provide data for all 5 rounds of the panel were considered lost to follow-up. In total, n=347 (approximately 3%) were lost to follow-up. Most of those who were lost to follow-up became institutionalized or died during the survey period. In general, those who were lost to follow-up were older and less physically and mentally healthy than those who provided complete follow-up. We do not include a formal statistical comparison between the analytic sample and those who did not have complete follow-up because of the very small percentage they represent. However, we now include a more specific statement on potential biases and generalizability in the discussion (lines 514-519). In light of the reviewer’s concerns, we also examined potential differences between the final analytic sample and those who were excluded due to proxy responses on the SAQ – which represented a comparatively larger percentage of the sample. Similarly, these individuals tended to be older and report poorer physical and mental health. We have added these results in the body of the paper, and provided some additional consideration of these findings in the discussion.**

8. The results should only be reported to two decimal places in the tables.

**We have made these changes.**

9. The results identify a range of modifiable variables associated with pain-related healthcare expenditure, but can you be sure about the direction of the relationship with MSK conditions/cost of treatment? Please comment on this in the discussion.

**We feel confident in the direction of the relationship (positive or negative association) but agree that the temporal or causal relationships between modifiable variables and costs cannot be established in this study. This study is one of the first in many steps toward understanding if and how we can better manage individuals at risk for high costs related to musculoskeletal pain. As an initial step, we are particularly interested in the associative relationship between costs and modifiable variables, as they represent potential treatment targets in future prospective studies that are designed to better establish temporal and causal relationships. We have provided further discussion of this point in the manuscript (line 512-516).**

10. People who were insured had higher expenditure, but this suggests that having insurance facilitates healthcare access and funding. Can you comment on potential unmet treatment needs in those with low costs?

**This is a very good point. Although under-treatment was not focus of our paper, we strongly believe that unmet needs are a threat to high value care in this population. While we are unable to provide data that affirms unmet needs among those with low costs in this analysis, we have added this consideration to the discussion (lines 471-487).**

11. I agree with the reviewers that excluding those with no healthcare costs at follow up seems like an odd omission if you are seeking to understand the scope of the problem at a population level. Couldn't you combine those with none and low healthcare expenditure, or have an additional group with $0 costs?

**Thank you for the opportunity to clarify our methodology. First and most importantly - we only excluded individuals if they had no costs for musculoskeletal pain in Year 1. Having costs in Year 1 was essentially an “index event” for this cohort to see if our definition of “persistent costs” was met. The rationale was that we wanted to study a healthcare-seeking population, and this was the most reasonable way to identify individuals with pain who were actually seeking care. We did not exclude anyone simply because they didn’t have health care costs at follow-up (Year 2). We have tried to clarify this approach in the revised manuscript.**

**The population of interest in our study is those who have already sought care for musculoskeletal pain. We believe that including individuals who had no costs whatsoever in either year would be addressing a fundamentally different question; what drives people to seek care versus not seek care at all? While this is also an important question, it has significantly different policy and clinical implications, and strays from the main point of our analysis. Ultimately, we want this line of research to inform policies and clinical decision-making at the point of care. Our findings may have implications for broader population-based, pain-related health policy, but the focus of this study is on those who engage with the health system (and have cost data available for the year following their engagement to establish level of “persistence”). We have provided some discussion regarding the need to further examine – in separate studies – the factors that drive people to seek care or not seek care at all, which is a related but distinctly different question than what we pose in this study. We have also tried to better clarify the intent of this study in the introduction and discussion.**

Review Comments to the Author

Reviewer #1: General comments:
This is a very well-written manuscript representing a very important health care topic, high costs associated with management of musculoskeletal conditions. The use of the Medical Expenditures Panel Survey is an appropriate and efficient data source. The findings of the analysis are important for understanding of this problem. Of great importance is the finding that 4% of the population are responsible for 35% of total costs. Minor clarifications as specified below are requested.

**Thank you for the positive comments.**

Specific comments:

1. Need further elaboration of how days of work missed due to illness is a modifiable factor. This seems more like an outcome.

**This was a challenging factor to classify and the author team had extensive discussions on how to handle it. Like many of the factors in this study, the degree of modifiability is variable. In the worker’s compensation literature, days of missed work is a modifiable factor that is targeted through treatment or policy. It is fairly well established that people with musculoskeletal conditions that return to work sooner – even at a reduced job duty level often have less work-related disability enhanced social participation, improved mental health and reduced costs (Cullen et al., J Occup Rehabil, 2018). In this paper, we wanted to reinforce that theoretically days of work missed could be modified through treatment or policy in non-occupational settings too. The original manuscript included a discussion on the duality of this factor in the limitations section, but we have made the discussion more robust in this revision.**

2. What is meant regarding the population of the study representing a very large population (Page 15), 12.985 represents 150,792,698 individuals?

**MEPS is a population-based survey that can be used to make inferences about the US population. MEPS takes a stratified approach to surveying the population, with over-sampling for certain under-represented subgroups. As a result, the raw sample does not appropriately reflect the true distribution or composition of the US population. To adjust, individual observations in the sample are weighted for analysis. Therefore, while the analysis includes data on 12,985 individuals, those individuals represent approximately 150 million individuals in the US population after weighting. This is a common approach to reporting when using population-based surveys that employ stratified sampling methods, like MEPS.**

3. The mean and median costs for the low and medium expenditure groups seems surprisingly low. $23 for the low group wouldn’t even cover an office visits. For the medium groups, $1551 may be reasonable but even that seems low. Please comment on this – is it possible that pain-related care is coded as something else?

**We agree this is an important point of clarification. Many of the individuals in the LOW and MEDIUM groups experienced costs in Year 1, but no costs in Year 2. When summed and averaged, the large number of $0 costs in Year 2 skew the mean and median such that spending appears very low (and it is, on average). This reinforces known patterns in health care spending that are characterized by high episodic costs, often followed by long periods of no or minimal costs (Johnson et al., Health Aff. 2015). It is possible that these events do not capture all pain-related costs – which is a limitation of any analysis that uses primarily survey data. However, these were the care episodes identified using the condition-event crosswalk, which is the suggested method for identifying condition-related costs in MEPS.**

4. The BMI means seem low and not representative of the overweight and obesity levels of the US population. It would be interesting to see the distribution (%) within the CDC weight classifications.

**The mean BMI of the sample is approximately 29 kg/m^2^ which puts this sample in the overweight category, on average, and only 1 point away from the obese category. This average is higher than what the CDC reports (source: NHANES) is the average BMI for men (26.6) and women (26.5) in the US. CDC classifications (weighted percentile) for BMI in this study were: underweight 1.2%; normal or healthy weight, 29.2%; overweight, 33.0%; and obese, 36.4%. Approximately 70% of the sample was either overweight or obese, which is slightly higher than CDC estimates of the general US population (64%, source: NHANES).**

5. Do number of prescriptions in table 1 include pain medications and non-pain medications? Does this refer to individual prescriptions filled or number of unique drugs per subject?

**The number of prescriptions in Table 1 include any medication that was linked to a musculoskeletal diagnosis through the condition-event crosswalk. Medications could have been for pain primarily (e.g., NSAIDs or opioids) but may have been for other indications related to the musculoskeletal condition (e.g., muscle relaxants, psychotropic medication). Number of prescriptions refers to total number of prescriptions filled, which includes original fills and refills. It is not the number of unique drugs per subject. We have re-written the section describing prescription medication to better reflect the data collected.**

6. There is a large proportion of subjects that are not employed, 45%. Is days missed from work relevant? Is this internally valid? That is, for persons who are not employed are there reported days missed from work?

**According to 2012 data released by the CDC from the National Health Interview Survey (www.cdc.gov/nchs/nhis/nhis_2012_data_release.htm), approximately 66% of the adult US population was in the workforce, with 34% not in workforce. For the purposes of this analysis, being not employed also included those who were retired or disabled. Given the high prevalence of musculoskeletal conditions among older adults that are often retired, as well as the frequency in which disability that prevents work is pain-related, we are not too surprised to see this percentage higher than the overall US population. We would not expect a substantial number of individuals who were unemployed (or retired) to report days of work missed. Because we control for employment status in the multivariate models, we are not concerned about this issue significantly impacting validity of the results regarding missed work. However, we have added more context to these results, and now also include a revised proportion of days missed based on number of respondents reporting employment.**

7. It is surprising that such a large proportion of the overall (77%) and 55% of the high utilizers report excellent, very good and good physical health? It might be useful to examine this as more than just a dichotomy with excellent, very good and good vs. fair or poor.

**Approximately 25% of the HIGH group reported excellent or very good physical health, while that percentage was 49% and 51% for the MEDIUM and LOW groups, respectively. This question asked respondents to rate their physical health compared to others, which may have led to more favorable responses than if asked to rate their physical health generally. Per the reviewer’s suggestion, we assessed whether a 3 level variable (excellent/very good vs good vs fair/poor) would be any more informative in our analysis. Results were similar to the original analysis for both the partially-adjusted model (significant at each level) and fully-adjusted model (no significant results), so we decided to keep the variable as categorized to be consistent with other variables in the analysis, including self-reported mental health.**

8. Related to previous comment, with 25% of persons in the high utilizer group reporting missing one or more days of work and 17% overall seems low – is this skewed by the large proportion of persons not employed? Can this be compared to other national data on missed time from work?

**Please see response to prior comment. Among those individuals who report employment, the prevalence of one or more missed work days is approximately 31%. This rate can be compared to data from the National Health Information Survey in the US from 2012 that reported approximately 40% of the workforce experiencing lost work days. More recent NHIS data indicate a similar percentage of 38%. Our sample was older, on average, than the general US population. Although older working adults tend to have a higher average number of missed work days, the incidence of one or more missed work day is lower among older adults compared to the general population, which may help to explain the lower incidence of one or more missed work days in our sample. These comparisons are now reported and discussed in the results.**

Reviewer #2: This paper confirms the findings of previous studies in other countries with single payer systems that the highest resource users with musculoskeletal conditions represent a small proportion of people with the condition.

The paper conducts a statistical analysis of the factors associated with high resource use. The paper is well written and clear and put in the context of prior studies.

**Thank you for the positive comments**

1. The paper is somewhat let down by the implied interpretation of the findings. That is, that while many patients do not receive the most effective and evidence based care, that this is a particular issue for those who are the highest resource users and that using more "value-based" approaches would reduce the high proportion of expenditure on the highest usage group. However it cannot be concluded from this study that the highest resource users are the most likely to be receiving more inappropriate care than any other patients, nor that use of evidence based approaches would reduce the cost for this group.

   A more accurate assessment from this study might be:

   - Being in the poorest health and poor (as well as pain interfering most with the patient's life) is associated with being in the highest cost group, as noted by the authors
   - When pain persists so does the expenditure
   - Many patients in other studies have been shown to receive less that optimal care without a good evidence base
   - Access to more evidence based approaches may improve health outcomes for some patients
   - Use of the most effective care may or may not reduce cost - this is not clear from this study.
   - Pain is not a one-size-fits-all condition, thus the most effective treatment needs to be found for each patient. Patient -centred care is important as pain is often complex and multifaceted and difficult to treat effectively.

**We agree with the reviewer’s assessment and appreciate the detailed thoughts. As the reviewer suggests, we are unable to establish whether high cost care is low-value care, and we do not mean to suggest that. In value-based systems, those with persistently high costs – especially for pain conditions that we would not expect to have persistently high costs – represent logical initial targets for studying if and how we might be able to deliver the same or better outcomes at a lower cost (which would improve value based on the simple definition of value). But those with low costs might also experience low value care and unmet needs. In the revised manuscript, we have removed language that may imply high costs necessarily means low value, while focusing on conclusions that we can draw from this study.**

General comments include:

- Was the number of musculoskeletal conditions and number and type of co-morbidities considered in the analysis as predictors of high resource use?

**The number of musculoskeletal conditions and number of comorbidities (using the Charlson comorbidity index) were considered as separate variables in the partially-adjusted and fully-adjusted models. The Charlson comorbidity index uses a method of weighting comorbidities such that it accounts for both number and type of comorbid condition.**

- Why exclude patients <18 years of age, as chronic pain can start in childhood?

**Chronic pain can begin in childhood for many individuals. However, assessment methods, approaches to treatment, and health care utilization patterns for pediatric populations may differ compared to adults, making results of a study that combines adult and pediatric populations difficult to interpret. More practically, SAQ data were not available for anyone under the age of 18, which would require removing many important self-reported variables from the analysis. We have noted this limitation and need for future study in the revised manuscript.**

- Why exclude patients who reported a musculoskeletal condition but no related expenditure, they equally may have not been receiving the most appropriate care? Were these patients a lower income group with less access to care?

**Please see response to the Editor regarding this important point. Briefly, we were interested in a health care seeking population. We agree that those who do not seek care could have unmet health care needs. Alternatively, they may feel comfortable managing pain on their own, or have pain that does not significantly impact their daily life. We have included a more robust discussion of this issue in the revision.**

- Comorbidities - can more detail be given on what is included in the index as this is an important predictor of resource use?

**These were conditions in the Charlson comorbidity index. Conditions included in this index are now listed in the appendix.**

- The self-efficacy assumption seems to be that the highest cost group should be able to manage their own condition. However there is no evidence for this and it seems very unlikely that the sickest group could manage their own pain without qualified medical support, given finding the optimal treatment is challenging even for specialist pain physicians.

**The assumption we were working under regarding self-efficacy is that those with low self-efficacy are likely to have higher expenditures, largely due to reduced capabilities or confidence in managing their health conditions on their own. We agree that finding optimal treatment can be challenging, particularly for those who have other medical conditions.**

- p 11 lines 198 and 199 seems to imply that the highest cost users are likely abusing prescription pain medication, or at least this is more likely in the highest resource use group, but there is no evidence for this.

**Thank you for pointing this out. We did not mean to imply this, only that prescription medicine abuse – largely within the opioid epidemic context – has created more focused interest on those who have high levels of prescription medication use. We have re-worded this sentence and re-focused the discussion in this paragraph.**

- p 13 Why allocate patients with zero expenditure in year 2 as low or middle - why not use total expenditure over 2 years?

**Persistence of costs was an important aspect of this study. Using total expenditures over two years would not help us differentiate between, for instance, individuals that had very expensive care in one year but no subsequent spending, and those who had recurring high costs over multiple years. We were interested in identifying the latter for the reasons outlined in the introduction and methods. Someone who had no expenditures in Year 2, by definition for the purposes of this study, could not have persistence of HIGH costs, and were therefore defined by their Year 1 spending.**

- How much of expenditure is predicted by severity of pain alone?

**MEPS does not include a specific pain severity measure, as it relates to pain intensity. Pain interference is one measure of pain severity and it was included in the SF-12 survey of the SAQ. It was a significant factor in partially-adjusted (LOW: RR=5.10, MEDIUM=3.16) and fully-adjusted (LOW: 1.61) models.**
