# Supplementary material for: Factors associated with persistently high-cost health care utilization for musculoskeletal pain
Source: PLoS One. 2019 Nov 11;14(11):e0225125. doi: 10.1371/journal.pone.0225125 (PMC6844454; doi:10.1371/journal.pone.0225125)
Supplement: Attachment — Submitted filename: PONE_Response_Second_Final.docx [file pone.0225125.s009.docx]

PONE-D-19-18187R1
Factors Associated with Persistently High-Cost Health Care Utilization for Musculoskeletal Pain
PLOS ONE

**Dear Dr. Giummarra,**

**We thank the editor and reviewers for their detailed comments, questions and suggestions on our revised manuscript. We have addressed all comments below. Thank you for considering our revised manuscript for publication.**

Thank you for addressing nearly all comments raised from the first review of your submission. There were a couple of points that still need to be addressed. First, thank you for the explanation for why an ordinal model was not used, but can you please provide further information in the manuscript on whether the assumptions for a multinomial model were met (i.e., the IIA). Second, you have highlighted that you chose to compare the characteristics/biases of the respondents excluded due to proxy response vs those that were included, but I did not see these results reported in the results section of the paper.

**We have included a short discussion on model selection in the revised manuscript (lines 277-292). Briefly, the assumptions for both ordinal and multinomial models were not met, however we chose to report results of the multinomial model as this approach provided separate parameters associated with each category (moving from LOW to MEDIUM, and LOW to HIGH) and was more interpretable in the context of this study. We also now include formal comparisons between those with a proxy response to the SAQ and those who provided self-responses to the SAQ in S2 and S3 tables, as well as a brief discussion of those results on lines 332-340, and implications of excluding these individuals on lines 570-574.**

Additional Editor Comments (if provided):

Please move discussion of the results to the discussion (e.g., the proportion of unemployed respondents compared with the US population).

**Completed.**

Review Comments to the Author

Reviewer #1:

This is a very well-written manuscript representing a very important health care topic, high costs associated with management of musculoskeletal conditions.  The use of the Medical Expenditures Panel Survey is an appropriate and efficient data source.  The findings of the analysis are important for understanding of this problem.  Of great importance is the finding that 4% of the population are responsible for 35% of total costs.   A minor explanation/clarification as specified below is requested.

Though I do say that all previous comments have been requested, the issue of extrapolation of the study population to the larger population it is intended to represent would be helpful.

Specific comment/question:

Line 307 – How the 12,985 study subjects was identified is clearly described.  In line 307 it is stated that these subjects represent over 150 million unique individuals – please explain.  I asked this question on the initial review, also.  This same issue occurs in lines 333 and 335 when the high and low cost subsets are described.

**We recognize this explanation may not have been clear in the prior response, and hope to provide further clarity here and in the revised manuscript. MEPS is designed as a population-based survey, the results of which can be used to make inferences about the general population within the United States. Like many population-based surveys, each respondent is meant to represent multiple individuals that comprise the entire population. However, MEPS employs a complex surveying design, one component of which includes over-sampling of certain under-represented population subgroups to ensure that variability within those subgroups is adequately reflected in the survey. Due to this complex surveying methodology, some respondents may represent more individuals in the population than others. Therefore, each respondent is assigned a survey weight – a numeric value that is used to weight their data for analysis so that analysis findings most accurately represent what we might expect to find if we were able to sample the entire US population. Because of this sampling methodology, the actual sample size (n=12,985) has limited relevance until it is properly weighted so we know how many people (and by extension what proportion of the US population with pain) these numbers actually represent.**

**Therefore, we report both the sample size and the overall number of individuals this sample represents across the study timeframe – as well as the number represented by the high and low costs groups. The use of population-based surveys to make population inferences, and the associated methodology and reporting is complex, although fairly standard and widely-published. Due to the complexity of the methods, we refer readers to more in-depth details on weighting and inference in the paper, but have tried to clarify how these samples are used to represent larger populations in the revised manuscript (lines 54-56, 327-332, 345-351, and 551-555).**

Reviewer #2: 
This is a well written paper on the higher service use group with musculoskeletal pain. I have a number of comments:

Overall: It is not surprising if people in the most pain and with the greatest pain interference use more health services. My main concern with the paper is the implicit suggestion that the high service use group should be using less services. However the paper does not contain the required information to determine whether the services used comply with current guidelines or best meet an individual patient’s pain management needs. It is important to remember that for some types of pain e.g. neuropathic pain, patients report a quality of life worse than death. See for example: Torrance et al (2014) Pain 155(10):1996-2004 doi: 10.1016/j.pain.2014.07.001. The biggest challenge in the field of chronic pain is the lack of effective treatment for many patients and the lack of access to the most effective treatments for others. This needs to be drawn out in the paper.

**We agree with the reviewer that the study does not evaluate whether services used were guideline-adherent, or whether they met the patient’s pain management needs. We disagree, however, that we are implicitly suggesting that the high service group should be using less services. In the revised manuscript, we have added a separate paragraph early in the discussion (439-448) that makes it explicitly clear that we are not suggesting the high service use group should unquestionably be using fewer services. Instead, our intent is to identify characteristics that are associated with persistence of high cost use so that we can study (in future analyses) whether or not there are opportunities to reduce utilization or costs, provide better outcomes and/or improve value of care for these individuals.**

**For better or for worse, the care provided to patients with persistently high costs is often scrutinized in value-based health care models – which are becoming more common in the US. Musculoskeletal conditions provide an appropriate population to study the complex issues of implementing value based care because they are highly prevalent and have a sufficient evidence based for determining which treatments may be considered guideline adherent. We hope to aid these patients by first understanding who they are (the aim of this study) and then determining if they are receiving or have access to the most effective care (the aim of future studies). We also agree with the reviewer that lack of effective treatments and lack of access are challenges to effective chronic pain management. However, as the reviewer cautions, we can only make inferences or suggestions based on our data. In this case, we cannot infer any more confidently that patients lack access to effective treatment than we can infer that they should be using fewer services. We have highlighted the potential for lack of access and need for more effective treatments more generally (lines 525-538), but refrain from drawing out this discussion for the reasons listed.**

Specific comments:

Suggest replacing the use of the word persistent with a more neutral term such as continuous. Persistent has a connotation that the patients are a nuisance.

**We assume the reviewer is referencing persistence as it relates to high costs or spending, and not as it relates to pain (i.e., persistent pain). We have very deliberately chosen the term persistent to be consistent with a fairly robust literature that uses this term to describe health care costs – specifically high costs – over time (see Yoon et al., Health Serv Res 2018; Figueroa et al., Ann Intern Med, 2018 for examples). We believe this will avoid confusion among readers and other investigators that might wish to directly compare our results to other studies.**

Methods:

Page 6 line 72: What are the conditions where expenditures are not expected to be persistently high and how was this definition arrived at? The severity and duration of pain is not uniform or easily predictable.

**Thank you for the opportunity to clarify. These conditions were listed in the supplemental appendix (S1 Appendix). Conditions were selected for exclusion based largely on whether they would clearly lead to high costs, especially persistence of high costs, such as amputation, traumatic wounds, spinal cord injury, congenital abnormalities and crushing injuries. To the reviewer’s point, some conditions included in the analysis could lead to severe pain of long duration, but we wouldn’t necessarily expect that in the majority of cases. For those that could be reasonably classified as either, we tried to err on the side of inclusion rather than exclusion to ensure better generalizability. The final list was agreed upon by a group of pain experts that included the author team. We have added further detail to this section in the revised manuscript (lines 79-82).**

P 7 lines 96-98 Are these expenditures specifically to treat chronic pain?

**These expenditures are to treat the musculoskeletal conditions to which they are linked. Some of those conditions may have been chronic, others may not have been chronic. We have provided some additional clarification in the expenditure summary section.**

P 10 lines 157 – 162 Asking patients if they feel they can overcome their illness without help from a medically trained person is rather odd in this context. Why should patients be able to do this when many clinicians find managing pain effectively difficult? Would the authors suggest that cancer or CVD patients should be able to manage their health condition without help from a medically trained person?

**Developing self-efficacy for managing one’s own pain is an important goal of effective chronic pain management (and possibly chronic pain prevention). Indeed, many individuals may never seek care, or seek less care, because they are able to effectively manage pain on their own. With this question, we were interested in evaluating a general “confidence in self-management” trait to determine whether it was associated with persistence of costs for musculoskeletal pain. The question was not specific to managing pain, but rather related to more general illness. As such, we felt it could give us insight into general self-efficacy for managing one’s own health. We have tried to provide better context around this question in the methods (lines 167-170). Regarding the comment on how this applies to other conditions, this is beyond the purposes of this current study as our focus was on musculoskeletal conditions. However self-efficacy is an important component of successful management of many conditions. Treatment of metastatic cancer may be less amenable to self-management, although sequela for cancer survivors like fatigue or psychological distress are amenable to self-management, and certainly many aspects of CVD could be self-managed through exercise, diet, changes in behavior, etc.**

P 12 lines 207-8 Use of opiods: There is now quite a large body of literature on overcoming medication nonadherance (also known as noncompliance)? Why is adherence to medical advice seen as a poor outcome in this paper?

**The authors do not view adherence to medical advice as a poor outcome and could not find any instances of that statement or implication in the paper. We do believe that some medical advice for pain management, at times, may lead to unnecessary treatment, low value and poor outcomes; but we do not promote non-adherence to medical advice. In the referenced statement, we note that the ongoing opioid crisis has caused health care systems, governing bodies, professional associations, providers, and patients to take a closer look at how exclusive use of pharmacological management for painful conditions contributes to both clinical outcomes and costs. This prompted the inclusion of this variable in our analysis. We are also careful not to suggest that any outcome is manifestly poor, only that there are certain outcomes, like persistence of high costs, that require further study to determine if these patients are receiving optimal care.**

Page 14 The high use group is the top 15% but the abstract refers to them as the top 4%. Can the authors please clarify?

**The high group was defined by persistence of costs in the top 15^th^ percentile of expenditures, but the number of individuals in that group represented 4% of the population studied. The 15% refers to percentile costs, the 4% refers to number or proportion of individuals.**

Results:
P 17 line 315 Does unemployed also include retired and not in the labour force for other reasons?

**Yes. We have added this detail to the referenced section (now lines 524-530).**

P 17 lines 319-330 This is confusing. Is the conclusion that patients in chronic pain take fewer days off work, and those who are older and in pain take even fewer days off work? If so, this is a remarkable and important conclusion that should be drawn out in the discussion. Were patients not in the labour force excluded from this analysis? Were the results adjusted for age?

**We agree this addition is not clear and have revised the wording in this section. Here we were trying to explain two findings that contrasted with previously-reported US population-based data on employment.**

**First, we observed a higher proportion of unemployed individuals in our sample compared to the average US population. We attributed this higher proportion to the older average age of the sample (i.e., more retirees), and the fact that those with pain conditions might be more likely to be out of work.**

**Second, we found a lower prevalence of missed work days than the US population average. At face value, this did not make sense because we would expect more work days missed among those with pain than those in the general population. However, an important nuance is that, on average, older adults in the general US population tend to have a lower incidence of missed work days than their younger counterparts. Therefore, we speculate that one reason for the low incidence of missed work days is that because our sample is older, on average, than the general US population, it is comprised of a larger proportion of individuals that are less likely to miss work. This is now clarified in on lines 501-520.**

**When all respondents were included, the rate of missed work days was 17%. When only those who were in the workforce were considered, the rate was 31%. All multivariable analyses were adjusted for age.**

P 22 lines 358-368 Highest use of services is amongst those who report the poorest physical and mental health – isn’t that to be expected? Was pain severity considered? How exactly is poorer health to be modified?

**This finding was not surprising. Unfortunately, and as mentioned in the prior revision response, pain severity measures are not included in MEPS as they pertain to pain intensity. Another measure of pain severity, pain interference, is documented in MEPS and was included in the analysis.**

**Poor physical or mental health could be modified in countless ways. Improved health might require changes in behavior, like participating in exercise, cessation of smoking, improving diet, changing jobs or joining social groups. It might require additional medical interventions like beginning pharmacological management for depression, anxiety, diabetes, or COPD, or seeking out psychological therapies. Methods for improving quality of life, including physical or mental health, are highly multidimensional and individual.**

Discussion:

P 27 While the treatments suggested may be beneficial, the median age of the sample for this study is 54 years with 44% unemployed. What treatments would benefit the group not in employment?

**We list a number of treatment targets on lines 449-453, all of which would be applicable to both a working and non-working population. If individuals were not employed because of their pain condition, treatments might focus on improving self-efficacy for pain management, graded exposure to work-related tasks, work re-integration, general physical conditioning, or cognitive behavioral theory-based strategies to improve pain coping or acceptance (lines 453-458). Given the scope of this study, we are careful to limit detailed suggestions for treatment.**

P 28 “Downstream effects of high prescription use”: The text implies that high use of medication causes the downstream effects - this needs to be more clearly described as an association not effects.
Perhaps the greatest need in relation to improved care of musculoskeletal pain conditions is for effective treatments for chronic pain to be accessible and affordable.

**We agree and have changed this wording.**
